# Supplementary material for: Mutual modulation of gut microbiota and the immune system in type 1 diabetes models
Source: Nat Commun. 2023 Nov 27;14:7770. doi: 10.1038/s41467-023-43652-x (PMC10682479; doi:10.1038/s41467-023-43652-x)
Supplement: Supplementary file 1 — Supplementary Information [file 41467_2023_43652_MOESM1_ESM.pdf]

SUPPLEMENTARY INFORMATION

Mutual modulation of gut microbiota and the immune system in type 1 diabetes models

Estela Rosell-Mases[1], Alba Santiago[2], Marta Corral-Pujol[1], Francisca Yáñez[2], Encarna Varela[2], Leire Egia-Mendikute[1], Berta Arpa[1], Catalina Cosovanu[1], Anaïs Panosa[3], Gerard Serrano-Gómez[2], Conchi Mora[1], Joan Verdaguer[1,4,#,*], and Chaysavanh Manichanh[2,5,#,*]

[1]Immunology and Immunopathology Group, Department of Experimental Medicine, Faculty of Medicine, Universitat de Lleida (UdL) and Institut de Recerca Biomèdica de Lleida (IRBLleida), 25198 Lleida, Spain.

[2]Microbiome Lab, Vall d'Hebron Institut de Recerca (VHIR), Vall d'Hebron Barcelona Hospital Campus, 08035 Barcelona, Spain.

[3]Flow Cytometry Facility, Universitat de Lleida (UdL) and Institut de Recerca Biomèdica de Lleida (IRBLleida), 25198 Lleida, Spain.

[4]CIBER of Diabetes and Associated Metabolic Diseases (CIBERDEM), Instituto de Salud Carlos III (ISCIII), 28029 Madrid, Spain.

[5]CIBER of Hepatic and Digestive Diseases (CIBERehd), Instituto de Salud Carlos III (ISCIII), 28029 Madrid, Spain

[#] These authors contributed equally.

[*]Corresponding authors: cmanicha@gmail.com (C.M.), joan.verdaguer@udl.cat (J.V.).

Supplementary Figures

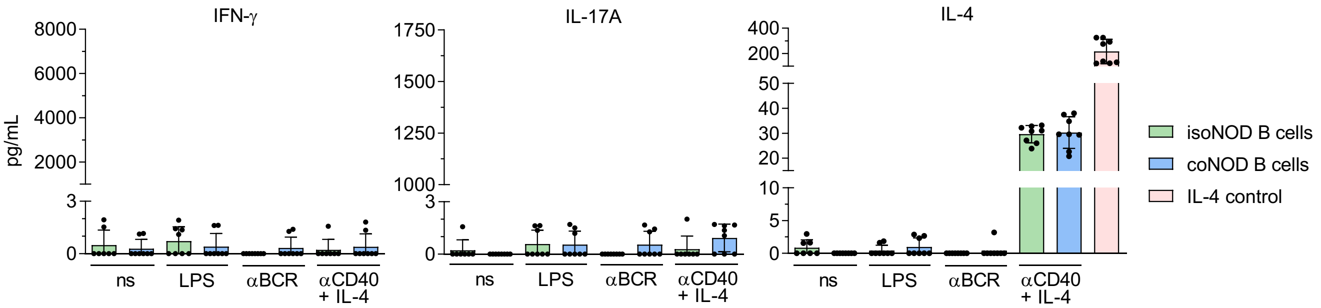

Supplementary Fig. 1 | *In vitro* secretion of IFN-γ, IL-17A, and IL-4 by B lymphocytes from NOD mice isolated and cohoused with 116C-NOD mice. B cells from NOD mice isolated (isoNOD B cells) and cohoused (coNOD B cells) with their 116C-NOD transgenic counterparts were cultured under different conditions: without stimulus (ns) (isoNOD: n=7, coNOD: n=8), with lipopolysaccharide (LPS) (isoNOD and coNOD: n=8), with anti-B cell receptor (αBCR) (isoNOD and coNOD: n=8), and with anti-CD40 (αCD40) plus IL-4 (isoNOD: n=7, coNOD: n=8). IL-4 control (n=8): wells without cells and with the same IL-4 concentration (to assess IL-4 consumption by B cells). Two independent experiments were performed (both shown). Data are expressed as mean±SD and analysed with two-way ANOVA test (two-sided) on rank-transformed data-values.

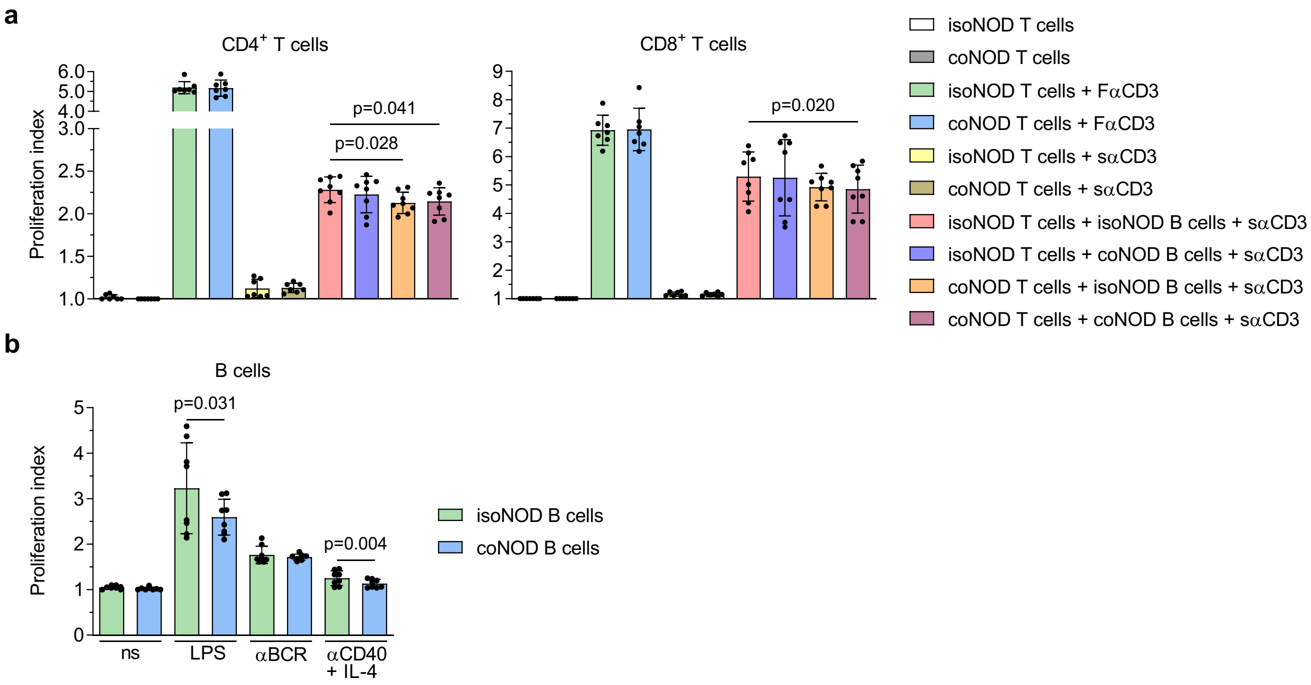

Supplementary Fig. 2 | Proliferation index of CD4$^+$ and CD8$^+$ T cells, and B cells from NOD mice isolated and cohoused with 116C-NOD mice. a T cells from NOD mice isolated (isoNOD T cells) and cohoused (coNOD T cells) were cultured *in vitro* under different conditions: alone (n=7 for isoNOD and coNOD), with well-coated or fixed anti-CD3 (FαCD3) (n=7 for isoNOD and coNOD), in the presence of soluble anti-CD3 (sαCD3) (n=7 for isoNOD and coNOD), and co-cultured with B cells from NOD mice isolated (isoNOD B cells) and cohoused (coNOD B cells), in their four possible combinations, plus sαCD3 (n=8 for each culture condition). **b** B cells from NOD mice isolated (isoNOD B cells) and cohoused (coNOD B cells) with their 116C-NOD transgenic counterparts were cultured under different conditions: without stimulus (ns), with lipopolysaccharide (LPS), with anti-B cell receptor (αBCR), and with anti-CD40 (αCD40) plus IL-4 (n=8 for isoNOD and coNOD B cells under each culture condition). Two independent experiments were conducted (both shown). Data are expressed as mean±SD and analysed with two-way ANOVA test (two-sided) on rank-transformed data-values.

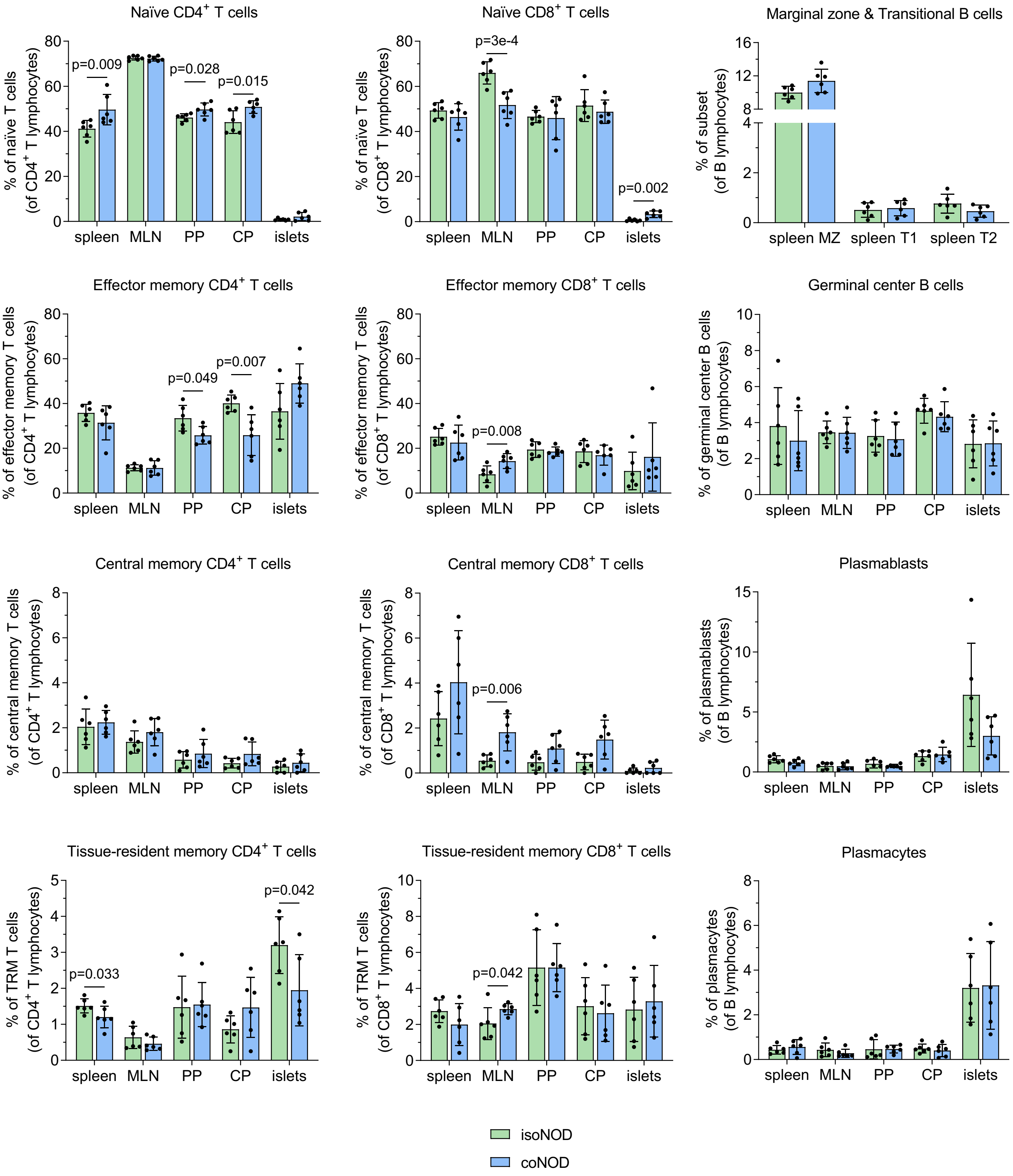

Supplementary Fig. 3 I T and B cell subsets of secondary lymphoid organs and pancreatic islets infiltrate from NOD mice isolated and cohoused with 116C-NOD mice. Direct *ex vivo* immunophenotyping of lymphocyte subpopulations within spleen, mesenteric lymph nodes (MLN), Peyer's patches (PP), cecal patch (CP) and pancreatic islet infiltrate, in NOD mice isolated (isoNOD) and cohoused (coNOD) (n=6 for each organ and group of mice). The $CD4^+$ and $CD8^+$ T cell subsets included: naïve T cells ($CD44^{low}$ $CD62L^+$ $CD69^-$), effector memory T cells ($CD44^{high}$ $CD62L^-$ $CD197^-$), central memory T cells ($CD44^{high}$ $CD62L^+$ $CD197^+$), and tissue-resident memory T cells ($CD44^{high}$ $CD62L^-$ $CD197^-$ $CD103^+$). The B cell subsets comprised: marginal zone B cells ($CD19^+$ $B220^+$ $CD93^-$ $CD21^{high}$ IgM^{high} IgD^{low} $CD23^-$), T1 B cells ($CD19^+$ $B220^+$ $CD93^+$ IgM^{high} $IgD^{+/-}$ $CD23^-$), T2 B cells ($CD19^+$ $B220^+$ $CD93^+$ IgM^{high} $IgD^{+/-}$ $CD23^+$), plasmablasts ($CD19^+$ $B220^+$ $CD138^+$), and plasmacytes ($CD19^-$ $B220^+$ $CD38^{low}$ $CD138^+$). Two independent experiments were performed (both shown). Data are expressed as mean±SD and analysed with two-way ANOVA test (two-sided) on rank-transformed data-values.

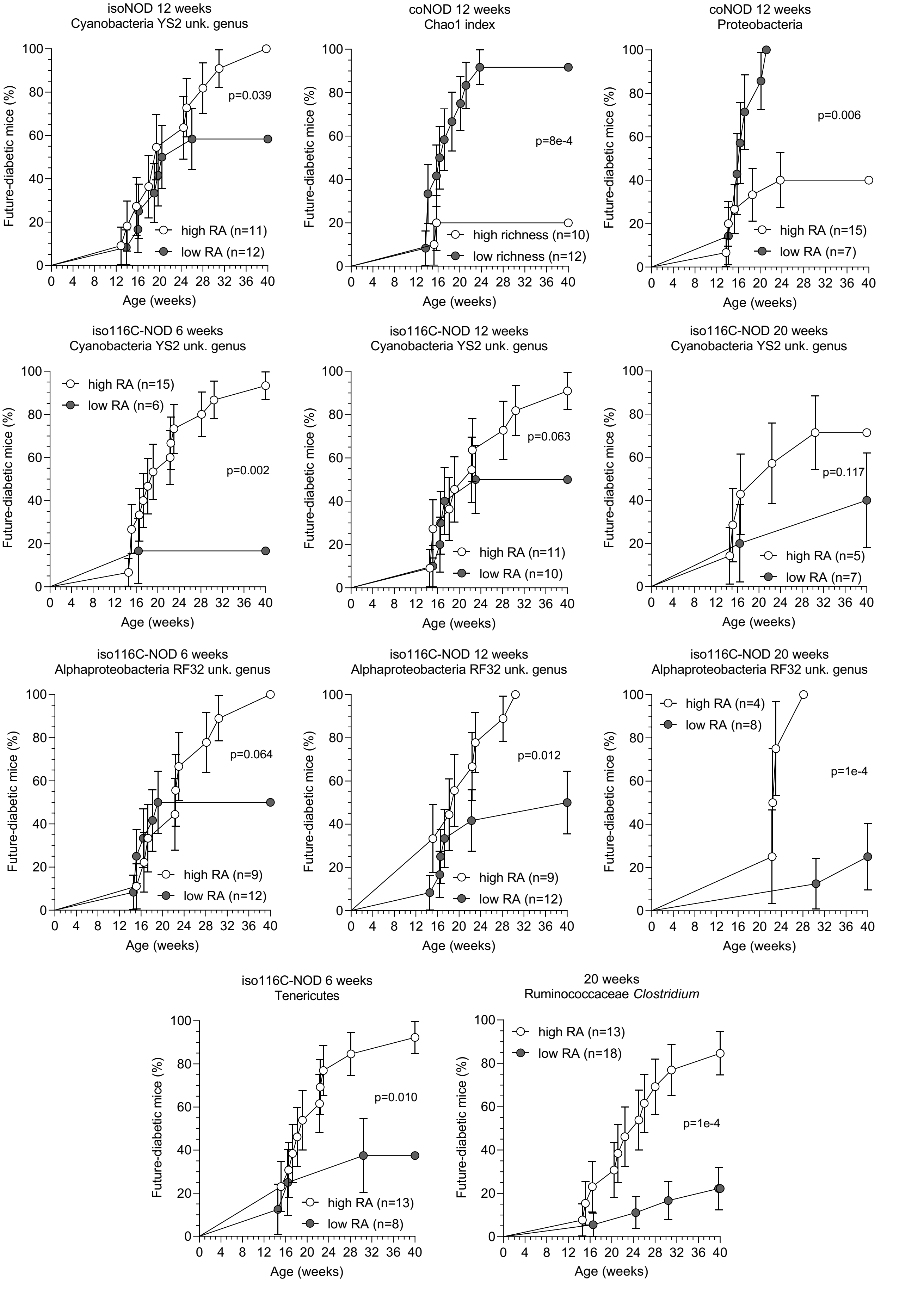

Supplementary Fig. 4 I Future T1D incidence of NOD and 116C-NOD mice classified by the relative abundance of gut bacterial taxa and level of richness. Future-diabetic and future-resistant isolated NOD (isoNOD), cohoused NOD (coNOD) and isolated 116C-NOD (iso116C-NOD) were divided into two subgroups: mice with high/low relative abundance (RA) of the corresponding bacterial taxa or high/low richness (Chao 1 index). Diabetes incidence curves were analysed with the Log-rank (Mantel-Cox) test (one-sided). Data are expressed as mean±SE.

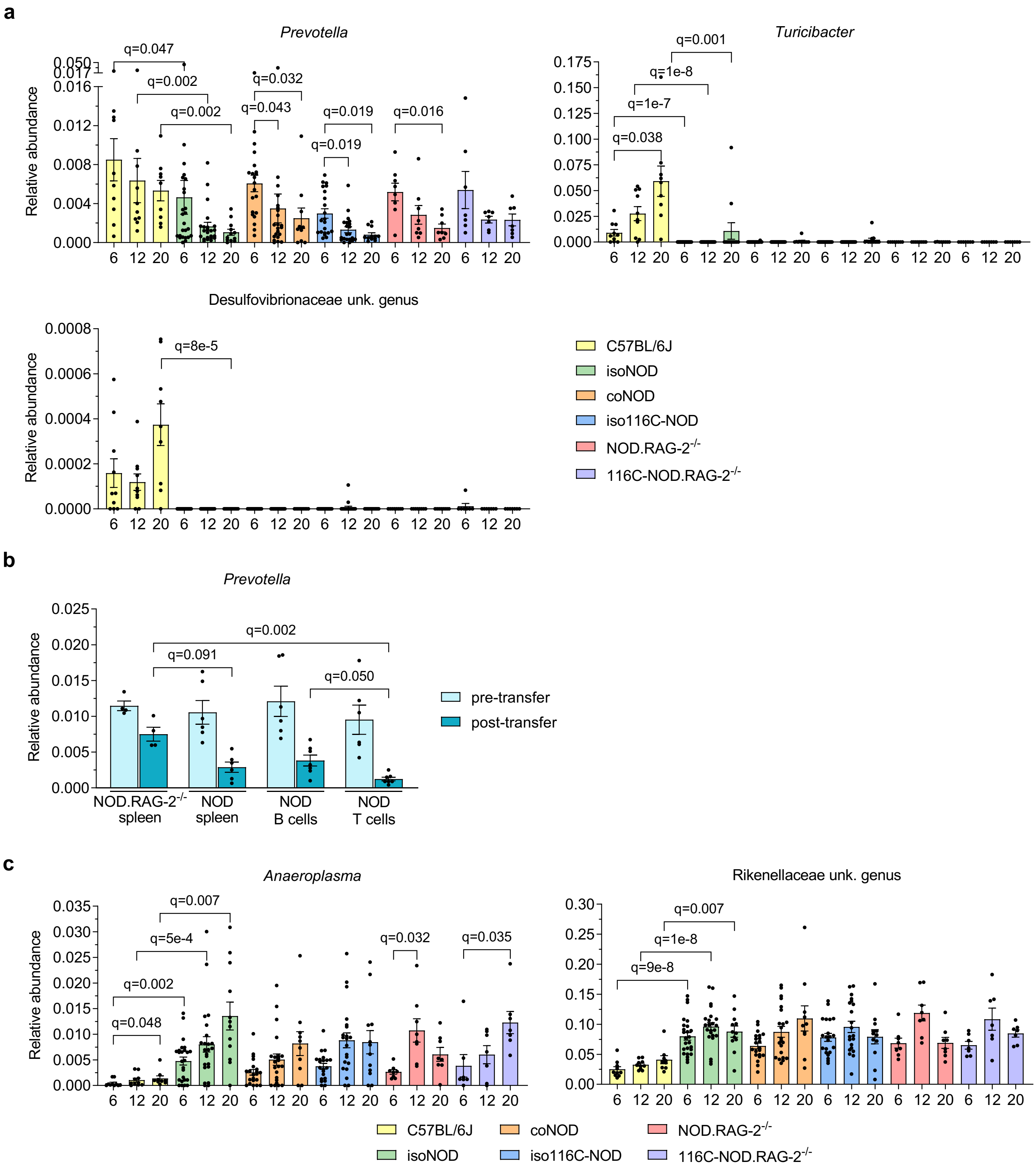

Supplementary Fig. 5 | Relative abundance of significant gut microbiota associated with genetic resistance and predisposition to autoimmune diabetes. 16S rRNA gene analysis was performed in faecal samples at 6, 12, and 20 weeks of age of different mouse strains: control C57BL/6J (6 weeks: n=10, 12 weeks: n=10, 20 weeks: n=9), isolated NOD or isoNOD (6 weeks: n=26, 12 weeks: n=23, 20 weeks: n=12), cohoused NOD or coNOD (6 weeks: n=21, 12 weeks: n=21, 20 weeks: n=10), isolated 116C-NOD or iso116C-NOD (6 weeks: n=21, 12 weeks: n=21, 20 weeks: n=12), NOD.RAG-2$^{-/-}$ (6 weeks: n=8, 12 weeks: n=8, 20 weeks: n=8), and 116C-NOD.RAG-2$^{-/-}$ (6 weeks: n=7, 12 weeks: n=7, 20 weeks: n=7). **a** Relative abundance of taxa associated with T1D resistance. **b** Relative abundance of *Prevotella* before (pre-transfer, 6 weeks) and after the transfer (post-transfer, 12 weeks) of total NOD spleen (n=6), NOD B cells (n=7), NOD T cells (n=7), and control NOD.Rag2$^{-/-}$ spleen (n=4). **c** Relative abundance of taxa related to T1D predisposition. Note: Rikenellaceae unknown (unk.) genus refers to Rikenellaceae;g__ in the database. Data are expressed as mean±SE and were analysed using the MaAsLin2 statistical framework (mixed-effects linear regression model, two-sided test, adjustment for multiple comparisons), where p-values were corrected using the false discovery rate (FDR).

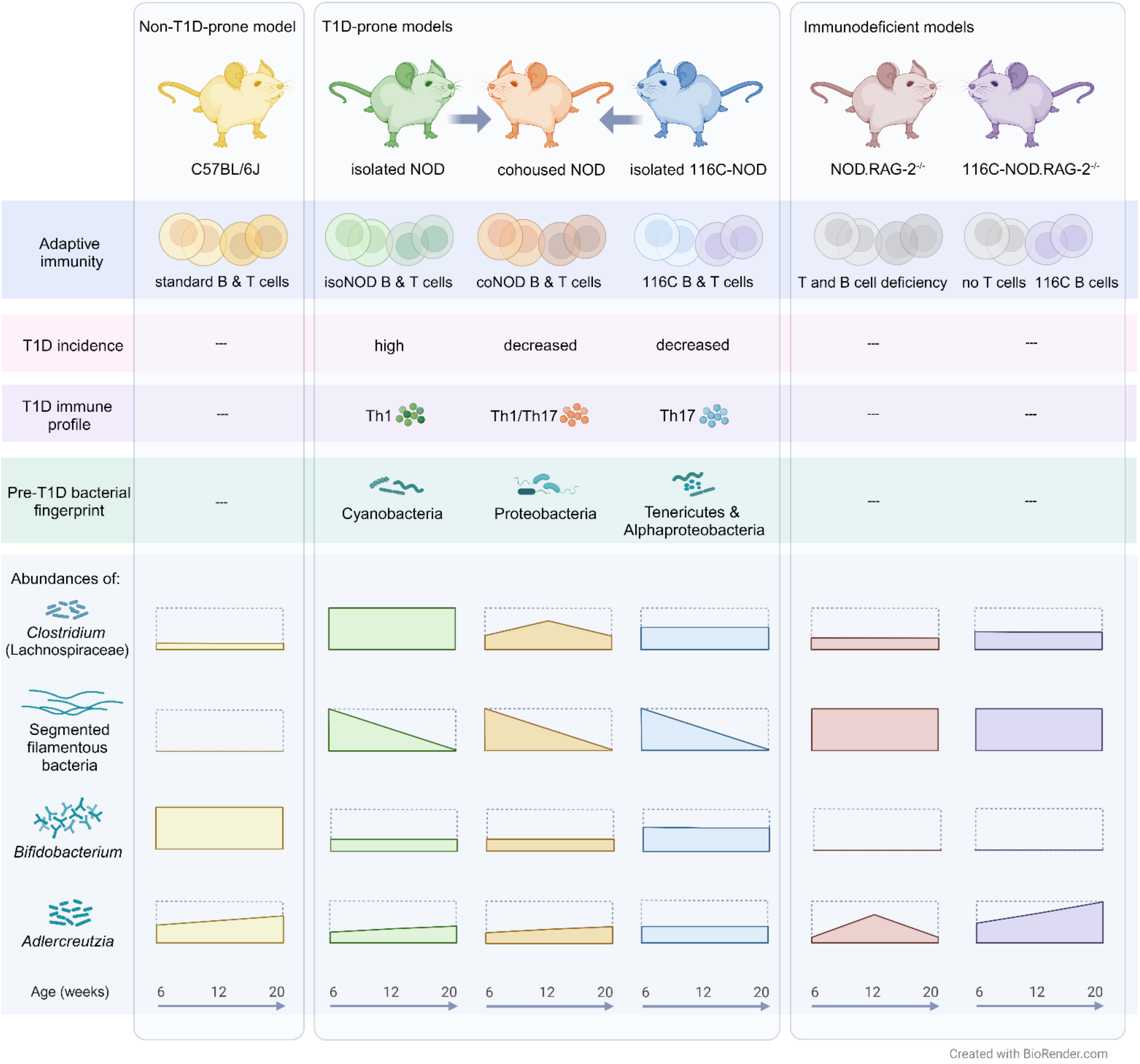

Supplementary Fig. 6 | Summary of the key features of the mouse models and the main results of the study. Created with BioRender.com.

Supplementary Tables

Supplementary Table 1. List of materials and resources.		
**Material or Resource**	**Source**	**Identifier**
Antibodies		
Purified Hamster Monoclonal Anti-Mouse CD3e (clone 145-2C11)	BD Pharmingen	Cat#553057 RRID: AB_394590
AffiniPure F(ab')$_2$ Fragment Donkey Polyclonal Anti-Mouse IgM, μ chain specific	Jackson Immunoresearch	Cat#715-006-020 RRID: AB_2340760
Purified Rat Monoclonal Anti-Mouse CD40 (clone 3/23)	BD Pharmingen	Cat#553787 RRID: AB_395051
VioletFluor 450 Rat Monoclonal Anti-Mouse CD19 (clone 1D3)	Tonbo Biosciences	Cat#75-0193-U100 RRID: AB_2621940
FITC Rat Monoclonal Anti-Mouse CD3 Molecular Complex (clone 17A2)	BD Pharmingen	Cat#561798 RRID: AB_395698
PerCP Rat Monoclonal Anti-Mouse CD4 (clone RM4-5)	BD Pharmingen	Cat#553052 RRID: AB_394587
PE Rat Monoclonal Anti-Mouse CD8a (clone 53-6.7)	BD Pharmingen	Cat#553033 RRID: AB_394571
PE-Cy7 Mouse Monoclonal Anti-Mouse T-bet (clone 4B10	eBioscience	Cat#25-5825-82 RRID: AB_11042699
Alexa Fluor 488 Rat Monoclonal Anti-Mouse GATA3 (clone TWAJ)	eBioscience	Cat#53-9966-42 RRID: AB_2574493
APC Rat Monoclonal Anti-mouse RORγT (clone AFKJS-9)	eBioscience	Cat#17-6988-82 RRID: AB_10609207
EFluor 450 Rat Monoclonal Anti-Mouse FOXP3 (clone FJK-16s)	eBioscience	Cat#48-5773-82 RRID: AB_467576
EFluor506 Rat Monoclonal Anti-Mouse CD8 (clone 53-6.7)	eBioscience	Cat#69-0081-82 RRID:AB_2637161
APC Rat Monoclonal Anti-Mouse CD62L (clone MEL-14)	BD Pharmingen	Cat#561919 RRID:AB_10895379
BV421 Rat Monoclonal Anti-Mouse CD44 (clone IM7)	Biolegend	Cat#103039 RRID:AB_10895752
PE Armenian Hamster Monoclonal Anti-Mouse CD69 (clone H1.2F3)	eBioscience	Cat#12-0691-81 RRID:AB_465731
BB515 Rat Monoclonal Anti-Mouse CD25 (clone PC61)	BD Pharmingen	Cat#564458 RRID:AB_2738814
PE-Cy7 Rat Monoclonal Anti-Mouse CD197 (clone 4B12)	Biolegend	Cat#120123 AB_2616687
APC-Cy7 Armenian Hamster Monoclonal Anti-Mouse CD103 (clone 2E7)	Biolegend	Cat#121431 AB_2566551
BV421 Armenian Hamster Monoclonal Anti-Mouse PD-1 (clone J43)	BD Pharmingen	Cat#562584 AB_2737668
APC-Fire750 Rat Monoclonal Anti-Mouse LAG-3 (clone C9B7W)	Biolegend	Cat#125240 AB_2876449
Efluor450 Rat Monoclonal Anti-Mouse FOXP3 (clone FJK-16s)	eBioscience	Cat#48-5773-82 AB_1518812
PE-Cy7 Rat Monoclonal Anti-Mouse CD73 (clone eBioTY/11.8 (TY/11.8))	eBioscience	Cat#25-0731-80 AB_10870789
APC Rat Monoclonal Anti-Mouse FR4 (clone 7D4)	BD Pharmingen	Cat#560318 AB_1645227
BV510 Rat Monoclonal Anti-Mouse CD19 (clone 6D5)	Biolegend	Cat#115545 AB_2562136
AlexaFluor 647 Rat Monoclonal Anti-Mouse B220 (clone RA3-6B2)	Biolegend	Cat#103226 AB_389330
PE-Cy7 Rat Monoclonal Anti-Mouse CD93 (clone AA4.1)	Biolegend	Cat#136505 AB_2044011
PE Rat Monoclonal Anti-Mouse CD21 (clone 7E9)	Biolegend	Cat#123409 AB_940411

AlexaFluor 488 Rat Monoclonal Anti-Mouse IgM (clone RMM-1)	Biolegend	Cat#406522<br>AB_2562859
PerCP Rat Monoclonal Anti-Mouse IgD (clone 11-26c.2a)	Biolegend	Cat#405736<br>AB_2563346
BV421 Rat Monoclonal Anti-Mouse CD23 (clone B3B4)	BD Pharmingen	Cat#562929<br>AB_2737898
APC-Fire750 Rat Monoclonal Anti-Mouse CD38 (clone 90)	Biolegend	Cat#102737<br>AB_2860597
BV421 Rat Monoclonal anti-Mouse CD138 (clone 281-2)	BD Pharmingen	Cat#566289<br>AB_2739663
PE-Cy7 Rat Monoclonal anti-Mouse GL-7 (clone GL7)	Biolegend	Cat#144619<br>AB_2800676
Chemicals		
Tissue Freezing Medium	Electron Microscopy Sciences	Cat#72592-C
Isopentane	Sigma-Aldrich	Cat#M32631
Hematoxylin 1-hydrate Gurr	VWR Chemicals	Cat#340374T
Aluminum sulfate octadecahydrate ($Al_2(SO_4)_3$)	VWR Chemicals	Cat#100103M
Sodium iodate	Honeywell Chemicals	Cat# 71702
Eosin Y Gurr	VWR Chemicals	Cat#341972Q
HBSS (Hank's Balanced Salt Solution)	Dutscher	Cat#X0509-500
FBS (Fetal Bovine Serum)	Gibco	Cat#10270106
RPMI 1640	Biowest	Cat#L0501-500
L-glutamine	Corning	Cat#25-005-CI
Sodium Pyruvate	Gibco	Cat#11360-070
2β-mercaptoethanol	Sigma-Aldrich	Cat#M6250-100ML
Benzylpenicillin sodium Penibiot “1”	Normon	Cat#602896.4
Streptomycin sulfate	Normon	Cat#624569.9
LPS (Lipopolysaccharides) from *Escherichia coli* O111:B4	Sigma-Aldrich	Cat#L3012-5MG
Recombinant Mouse IL-4 Protein	R&D Systems	Cat#404-ML-010/CF
Collagenase type IV	Worthington	Cat#LS004188
Guanidine thiocyanate	Sigma-Aldrich	Cat#G6639
N-lauryl sarcosine	Sigma-Aldrich	Cat#L9150
Critical commercial assays		
Mouse Pan T Cell Isolation Kit II	Miltenyi Biotec	Cat#130-095-130
Mouse B Cell Isolation Kit	Miltenyi Biotec	Cat#130-090-862
Cytometric Bead Array (CBA) Mouse Th1/Th2/Th17 Cytokine Kit	BD Pharmingen	Cat#560485
Foxp3/Transcription Factor Staining Buffer Set	eBioscience	Cat#00-5523-00
FITC-dextran 4 kDa (FD4)	TdB Labs	CAS#60842-46-8
CFSE CellTrace	Invitrogen	Cat#C34554
Deposited data		
16S data	NCBI database	Access number: PRJNA989542 [https://www.ncbi.nlm.nih.gov/bioproject/PRJNA989542]
Experimental models		
NOD mouse (original nomenclature: NOD/ShiLtJ)	The Jackson Laboratory (Bar Harbor, ME)	Cat#JAX:001976<br>RRID:IMSR_ARC:NOD
NOD.RAG-$2^{-/-}$ knockout immunodeficient mouse	Dr. P. Santamaria (University of Calgary, Alberta, Canada)	N/A
116C-NOD transgenic mouse	Carrascal *et al.*, 2016	N/A

116C-NOD.RAG-$2^{-/-}$ transgenic immunodeficient mouse	Carrascal *et al.*, 2016	N/A
C57BL/6J mouse	The Jackson Laboratory (Charles River, Europe)	Cat#JAX:000664 RRID:IMSR_ JAX:000664
Oligonucleotides		
V4F_515_19: 5'-GTGCCAGCAMGCCGCGGTAA-3'	Integrated DNA Technologies	Custom primers
V4R_806_20: 5'-GGACTACCAGGGTATCTAAT-3'	Integrated DNA Technologies	Custom primers
Software and algorithms		
FCAP Array Software v3.0	BD Biosciences	https://www.bdbiosciences.com/en-ca/products/instruments/software-informatics/instrument-software/fcap-array-software-v3-0.652099
FlowJo 10.0.7	BD Biosciences	https://www.flowjo.com
GraphPad Prism 9.0.0	GraphPad Software	https://www.graphpad.com
FCS Express 7.18.0015	De Novo Software	https://denovosoftware.com/
QIIME2		https://qiime2.org/
MaAsLin2		https://huttenhower.sph.harvard.edu/maaslin/
Animal House Materials		
Teklad Global 18% Protein Rodent Diet	Envigo	Cat#2018S
Medi-Test Glucose urine test strips	Macherey-Nagel	Cat#93001
Accu-Chek Performa Glucose blood test strips	Roche	Cat#06454011
Critical Instruments and Consumables		
AutoMACS Pro Separator	Miltenyi Biotec	Cat#130-092-545
BD FACSCanto II Flow Cytometer	BD Biosciences	
Nunclon Delta round-bottom 96-well plates	Nunc	Cat#163320
Immulon 4 HBX flat-bottom 96-well pates	Nunc	Cat#047612
K3 EDTA microtubes	Sarstedt	Cat#41.1395.005
Black flat-bottom 96-well plate (chimney)	Greiner Bio-One	Cat#655076
Infinite M200 fluorescence microplate reader	Tecan	
