# Supplementary material for: Mutual modulation of gut microbiota and the immune system in type 1 diabetes models
Source: Nat Commun. 2023 Nov 27;14:7770. doi: 10.1038/s41467-023-43652-x (PMC10682479; doi:10.1038/s41467-023-43652-x)
Supplement: Supplementary file 3 — Reporting Summary [file 41467_2023_43652_MOESM3_ESM.pdf]

nature portfolio

Corresponding author(s): Chaysavanh Manichanh, Joan Verdaguer

Last updated by author(s): 29/10/2023

Reporting Summary

Nature Portfolio wishes to improve the reproducibility of the work that we publish. This form provides structure for consistency and transparency in reporting. For further information on Nature Portfolio policies, see our Editorial Policies and the Editorial Policy Checklist.

Statistics

For all statistical analyses, confirm that the following items are present in the figure legend, table legend, main text, or Methods section.

n/a	Confirmed	
☐	☒	The exact sample size (*n*) for each experimental group/condition, given as a discrete number and unit of measurement
☐	☒	A statement on whether measurements were taken from distinct samples or whether the same sample was measured repeatedly
☐	☒	The statistical test(s) used AND whether they are one- or two-sided<br>*Only common tests should be described solely by name; describe more complex techniques in the Methods section.*
☒	☐	A description of all covariates tested
☐	☒	A description of any assumptions or corrections, such as tests of normality and adjustment for multiple comparisons
☐	☒	A full description of the statistical parameters including central tendency (e.g. means) or other basic estimates (e.g. regression coefficient) AND variation (e.g. standard deviation) or associated estimates of uncertainty (e.g. confidence intervals)
☐	☒	For null hypothesis testing, the test statistic (e.g. *F*, *t*, *r*) with confidence intervals, effect sizes, degrees of freedom and *P* value noted<br>*Give P values as exact values whenever suitable.*
☒	☐	For Bayesian analysis, information on the choice of priors and Markov chain Monte Carlo settings
☒	☐	For hierarchical and complex designs, identification of the appropriate level for tests and full reporting of outcomes
☒	☐	Estimates of effect sizes (e.g. Cohen's *d*, Pearson's *r*), indicating how they were calculated

Our web collection on statistics for biologists contains articles on many of the points above.

Software and code

Policy information about availability of computer code

Data collection	No software was used
Data analysis	FCAP Array Software v3.0, FlowJo 10.0.7, GraphPad Prism 9.0.0, QIIME v2.0, MaAsLin2, FCS Express 7.18.0015.

For manuscripts utilizing custom algorithms or software that are central to the research but not yet described in published literature, software must be made available to editors and reviewers. We strongly encourage code deposition in a community repository (e.g. GitHub). See the Nature Portfolio guidelines for submitting code & software for further information.

Data

Policy information about availability of data

All manuscripts must include a data availability statement. This statement should provide the following information, where applicable:

- Accession codes, unique identifiers, or web links for publicly available datasets
- A description of any restrictions on data availability
- For clinical datasets or third party data, please ensure that the statement adheres to our policy

All sequence data generated in this study have been deposited in the NCBI database with the following access number: PRJNA989542 [https://www.ncbi.nlm.nih.gov/bioproject/PRJNA989542]. The source data used in this study are available in the Figshare data repository [10.6084/m9.figshare.24459619].

Research involving human participants, their data, or biological material

Policy information about studies with human participants or human data. See also policy information about sex, gender (identity/presentation), and sexual orientation and race, ethnicity and racism.

Reporting on sex and gender	N/A
Reporting on race, ethnicity, or other socially relevant groupings	N/A
Population characteristics	N/A
Recruitment	N/A
Ethics oversight	N/A

Note that full information on the approval of the study protocol must also be provided in the manuscript.

Field-specific reporting

Please select the one below that is the best fit for your research. If you are not sure, read the appropriate sections before making your selection.

- [x] Life sciences
- [ ] Behavioural & social sciences
- [ ] Ecological, evolutionary & environmental sciences

For a reference copy of the document with all sections, see nature.com/documents/nr-reporting-summary-flat.pdf

Life sciences study design

All studies must disclose on these points even when the disclosure is negative.

Sample size	The sample sizes were the needed to perform statistical analyses. For a reliable assessment of diabetes incidence in AD-prone mice, the minimum sample size is around n=20. A sample size range of n=30-40 is the optimum.
Data exclusions	No data exclusions
Replication	The cytokine profiling, transcription factor analysis, proliferation assay, direct ex vivo immunophenotyping of lymphocyte subsets, and intestinal permeability analysis were conducted in two independent experiments (the total number of samples, including both experiments, is shown in each graphic and figure legend). The replication was successful.
Randomization	NOD and 116C-NOD mice were intended for isolation or cohousing conditions depending on the number of transgenic and non-transgenic mice obtained in each litter. The number of NOD and 116C-NOD mice was balanced in cohousing cages. Whenever necessary, groups of mice were randomly separated.
Blinding	Whenever possible, mice were handled under blinded conditions. All outcome analyses were performed blinded.

Reporting for specific materials, systems and methods

We require information from authors about some types of materials, experimental systems and methods used in many studies. Here, indicate whether each material, system or method listed is relevant to your study. If you are not sure if a list item applies to your research, read the appropriate section before selecting a response.

Materials & experimental systems

n/a	Involved in the study	
[ ]	[x]	Antibodies
[x]	[ ]	Eukaryotic cell lines
[x]	[ ]	Palaeontology and archaeology
[ ]	[x]	Animals and other organisms
[x]	[ ]	Clinical data
[x]	[ ]	Dual use research of concern
[x]	[ ]	Plants

Methods

n/a	Involved in the study	
[x]	[ ]	ChIP-seq
[ ]	[x]	Flow cytometry
[x]	[ ]	MRI-based neuroimaging

Antibodies

Antibodies used	Purified Hamster Monoclonal Anti-Mouse CD3e, clone 145-2C11 (BD Pharmingen, Cat#553057, RRID: AB_394590); AffiniPure $F(ab')_2$ Fragment Donkey Polyclonal Anti-Mouse IgM, µ chain specific (Jackson Immunoresearch, Cat#715-006-020, RRID: AB_2340760);

Purified Rat Monoclonal Anti-Mouse CD40, clone 3/23 (BD Pharmingen, Cat#553787, RRID: AB_395051); VioletFluor 450 Rat Monoclonal Anti-Mouse CD19, clone 1D3 (Tonbo Biosciences, Cat#75-0193-U100, RRID: AB_2621940); FITC Rat Monoclonal Anti-Mouse CD3 Molecular Complex, clone 17A2 (BD Pharmingen, Cat#561798, RRID: AB_395698); PerCP Rat Monoclonal Anti-Mouse CD4, clone RM4-5 (BD Pharmingen, Cat#553052, RRID: AB_394587); PE Rat Monoclonal Anti-Mouse CD8a, clone 53-6.7 (BD Pharmingen, Cat#553033, RRID: AB_394571); PE-Cy7 Mouse Monoclonal Anti-Mouse T-bet, clone 4B10 (eBioscience, Cat#25-5825-82, RRID: AB_11042699); Alexa Fluor 488 Rat Monoclonal Anti-Mouse GATA3, clone TWAJ (eBioscience, Cat#53-9966-42, RRID: AB_2574493); APC Rat Monoclonal Anti-Mouse RORɣT, clone AFKJS-9 (eBioscience, Cat#17-6988-82, RRID: AB_10609207); EFluor 450 Rat Monoclonal Anti-Mouse FOXP3, clone FJK-16s (eBioscience, Cat#48-5773-82, RRID: AB_467576). EFluor506 Rat Monoclonal Anti-Mouse CD8 (clone 53-6.7) (eBioscience, Cat#69-0081-82, RRID:AB_2637161); APC Rat Monoclonal Anti-Mouse CD62L (clone MEL-14) (BD Pharmingen, Cat#561919, RRID:AB_10895379); BV421 Rat Monoclonal Anti-Mouse CD44 (clone IM7) (Biolegend, Cat#103039, RRID:AB_10895752); PE Armenian Hamster Monoclonal Anti-Mouse CD69 (clone H1.2F3) (eBioscience, Cat#12-0691-81, RRID:AB_465731); BB515 Rat Monoclonal Anti-Mouse CD25 (clone PC61) (BD Pharmingen, Cat#564458, RRID:AB_2738814); PE-Cy7 Rat Monoclonal Anti-Mouse CD197 (clone 4B12) (Biolegend, Cat#120123, AB_2616687); APC-Cy7 Armenian Hamster Monoclonal Anti-Mouse CD103 (clone 2E7) (Biolegend, Cat#121431, AB_2566551); BV421 Armenian Hamster Monoclonal Anti-Mouse PD-1 (clone J43) (BD Pharmingen, Cat#562584, AB_2737668); APC-Fire750 Rat Monoclonal Anti-Mouse LAG-3 (clone C9B7W) (Biolegend, Cat#125240, AB_2876449); Efluor450 Rat Monoclonal Anti-Mouse FOXP3 (clone FJK-16s) (eBioscience, Cat#48-5773-82, AB_1518812); PE-Cy7 Rat Monoclonal Anti-Mouse CD73 (clone eBioTY/11.8 (TY/11.8)) (eBioscience, Cat#25-0731-80, AB_10870789); APC Rat Monoclonal Anti-Mouse FR4 (clone 7D4) (BD Pharmingen, Cat#560318, AB_1645227); BV510 Rat Monoclonal Anti-Mouse CD19 (clone 6D5) (Biolegend, Cat#115545, AB_2562136); AlexaFluor 647 Rat Monoclonal Anti-Mouse B220 (clone RA3-6B2) (Biolegend, Cat#103226, AB_389330); PE-Cy7 Rat Monoclonal Anti-Mouse CD93 (clone AA4.1) (Biolegend, Cat#136505, AB_2044011); PE Rat Monoclonal Anti-Mouse CD21 (clone 7E9) (Biolegend, Cat#123409, AB_940411); AlexaFluor 488 Rat Monoclonal Anti-Mouse IgM (clone RMM-1) (Biolegend, Cat#406522, AB_2562859); PerCP Rat Monoclonal Anti-Mouse IgD (clone 11-26c.2a) (Biolegend, Cat#405736, AB_2563346); BV421 Rat Monoclonal Anti-Mouse CD23 (clone B3B4) (BD Pharmingen, Cat#562929, AB_2737898); APC-Fire750 Rat Monoclonal Anti-Mouse CD38 (clone 90) (Biolegend, Cat#102737, AB_2860597); BV421 Rat Monoclonal anti-Mouse CD138 (clone 281-2) (BD Pharmingen, Cat#566289, AB_2739663); PE-Cy7 Rat Monoclonal anti-Mouse GL-7 (clone GL7) (Biolegend, Cat#144619, AB_2800676).

Validation	All the antibodies were validated for mouse specie. Purified Hamster Monoclonal Anti-Mouse CD3e, AffiniPure $F(ab')_2$ Fragment Donkey Polyclonal Anti-Mouse IgM and Purified Rat Monoclonal Anti-Mouse CD40 were validated for cell stimulation application. The remaining antibodies were validated for flow cytometry application. All antibodies were used following the recommendations described in the data sheet provided by the manufacturer.

Animals and other research organisms

Policy information about studies involving animals; ARRIVE guidelines recommended for reporting animal research, and Sex and Gender in Research

Laboratory animals	NOD mouse (original nomenclature: NOD/ShiLtJ), The Jackson Laboratory, Bar Harbor, ME, (Cat#JAX:001976, RRID:IMSR_ARC:NOD); NOD.RAG-2-/- knockout immunodeficient mouse (Dr. P. Santamaria, University of Calgary, Alberta, Canada); 116C-NOD transgenic mouse (Carrascal et al., 2016); 116C-NOD.RAG-2-/- transgenic immunodeficient mouse (Carrascal et al., 2016); C57BL/6J mouse, The Jackson Laboratory, Charles River, Europe (Cat#JAX:000664, RRID:IMSR_JAX:000664). Diabetes in mice from NOD (isolation, cohousing, and cage change groups), 116C-NOD, NOD.RAG-2-/- and 116C-NOD.RAG-2-/- strains was followed up for 40 weeks. Insulitis score was analyzed in NOD and 116C-NOD mice at 6 and 12 weeks of age. Immunological studies and gut permeability assay were performed at 12 weeks. Fecal samples from NOD mice, 116C-NOD, NOD.RAG-2-/-, 116C-NOD.RAG-2-/- and C57BL/6J mice were collected at 6, 12, and 20 weeks of age. NOD.RAG-2-/- females at 6 weeks of age were intravenously injected with lymphocytes from 6-week-old NOD donors (or NOD.Rag2-/- donors as controls), reaching the end of the study at 12 weeks of age.<br>Mice were bred and maintained in the rodent animal house of the UdL. Animals were kept under specific pathogen-free (SPF) conditions and provided with autoclaved food (Envigo, Cat#2018S) and water ad libitum. The light-dark cycle was controlled in a 12:12h format. Temperature was set at 21±2ºC, and relative humidity was held at 55±5%. All the mice cages were located in the same SPF room. None of the mice received antibiotic treatment. Anaesthesia was induced and maintained via isoflurane inhalation at 4% and 2%, respectively. Mice were euthanized either by isoflurane inhalation or cervical dislocation when they developed diabetes or at the end of the studies.
Wild animals	No wild animals were used in the study.
Reporting on sex	The experiments were developed in females, due to their higher T1D incidence compared to males.
Field-collected samples	No field-collected samples were used in the study.
Ethics oversight	Animal handling, maintenance and experimentation were conducted in accordance with the guidelines stated by the European Legislation for the Protection of Animals Used for Scientific Purposes. The animal procedures were approved by the Committee on the Ethics of Research in Animal Experimentation of the UdL.

Note that full information on the approval of the study protocol must also be provided in the manuscript.

Plants

Seed stocks	N/A
Novel plant genotypes	N/A
Authentication	N/A

Flow Cytometry

Plots

Confirm that:

- [ ] The axis labels state the marker and fluorochrome used (e.g. CD4-FITC).
- [ ] The axis scales are clearly visible. Include numbers along axes only for bottom left plot of group (a 'group' is an analysis of identical markers).
- [ ] All plots are contour plots with outliers or pseudocolor plots.
- [ ] A numerical value for number of cells or percentage (with statistics) is provided.

Methodology

Sample preparation	Spleens, MLNs, PP, CP and islets were mechanically disrupted with glass slide frosted ends in HBSS (Dutscher, Cat#X0509-500) containing 1% heat-inactivated fetal bovine serum or hiFBS (Gibco, Cat#10270106) and converted into

single-cell suspensions by passing splenocytes through 40 μm nylon filters. Please refer to the Methods section for further details.

Instrument	FACSCanto II (BD Biosciences, Cat#640806); AutoMACS Pro Separator (Miltenyi Biotec, Cat#130-092-545).
Software	FlowJo 10.0.7 (BD Biosciences, https://www.flowjo.com); FCAP Array Software v3.0 (BD Biosciences, https://www.bdbiosciences.com/en-ca/products/instruments/software-informatics/instrument-software/fcap-array-software-v3-0.652099).
Cell population abundance	T and B lymphocytes were separately purified via negative selection using isolation kits specific for each population: Mouse Pan T Cell Isolation Kit II (Miltenyi Biotec, Cat#130-095-130) and Mouse B Cell Isolation Kit (Miltenyi Biotec, Cat#130-090-862); as well as the AutoMACS Pro Separator magnetic cell sorter (Miltenyi Biotec, Cat#130-092-545), following manufacturer's instructions. Yield and purity of T and B cells were assessed by staining CD3 and CD19 cell surface markers with the monoclonal antibodies FITC anti-CD3 (BD Pharmingen, Cat#561798) at 2 μg/mL and violetFluor 450 anti-CD19 (Tonbo Biosciences, Cat#75-0193-U100) at 0.8 μg/mL in PBS with 1% hiFBS at 4ºC for 20 minutes, and by using the flow cytometer FACSCanto II (BD Biosciences, Cat#640806). Lymphocyte purity was only accepted when values were greater than 90%. From a sample of 1x10exp07 splenocytes, around 0.4x10exp07 T cells (60% CD4+, 40% CD8+ T lymphocytes) and 0.5x10exp07 B cells were obtained.
Gating strategy	Doublets were removed by using FSC-A/FSC-H gating. The viable lymphocytes were selected by the FSC-A/SSC-A profile.<br>The yield and purity assessment of T and B cells was analyzed by gating the CD3 and CD19 positive events, respectively, on the lymphocyte gate.<br>The percentages of T cell subsets, based on transcription factors analysis, were determined on the CD4 positive cells as follows. T-bet+, RORɣT+, GATA3+ and Foxp3+ subpopulations were analyzed by selecting the total of positive cells for the staining of Tbet, RORɣT, GATA3 and Foxp3, respectively. The T-bet+ RORɣT+ double positive subset was determined by gating the positive cells for Tbet / RORɣT and negative for GATA3 / Foxp3 stainings.<br>The assessed CD4+ and CD8+ T cell subsets, based on cell surface markers and intracellular Foxp3 staining, included: naïve T cells (CD44low CD62L+ CD69-), effector T cells (CD44high CD62L- CD69+ CD25+), effector memory T cells (CD44high CD62L- CD197-), central memory T cells (CD44high CD62L+ CD197+), tissue-resident memory T cells (CD44high CD62L- CD197- CD103+), exhausted-like T cells (PD-1(CD279)+) and LAG-3 (CD223)+), and anergic-like T cells (Foxp3- CD73high FR4high).<br>The evaluated B cell subsets encompassed the following: follicular B cells (CD19+ B220+ CD93- CD21low IgM+ IgDhigh CD23+), marginal zone B cells (CD19+ B220+ CD93- CD21high IgMhigh IgDlow CD23-), T1 B cells (CD19+ B220+ CD93+ CD21low IgMhigh IgD+/- CD23-), T2 B cells (CD19+ B220+ CD93+ IgMhigh IgD+/- CD23+), anergic B cells (CD19+ B220+ CD93- IgM- IgDhigh), germinal centre B cells (CD19+ B220+ CD38low CD138- GL-7+), memory B cells (CD19+ B220+ CD38high CD138- GL-7-), plasmablasts (CD19+ B220+ CD138+), and plasmacytes (CD19- B220+ CD38low CD138+).<br>Please refer to the Methods section for further details.

☐ Tick this box to confirm that a figure exemplifying the gating strategy is provided in the Supplementary Information.
